# Arginine Vasopressin Is a Blood-Based Biomarker of Social Functioning in Children with Autism

**DOI:** 10.1371/journal.pone.0132224

**Published:** 2015-07-22

**Authors:** Dean S. Carson, Joseph P. Garner, Shellie A. Hyde, Robin A. Libove, Sean W. Berquist, Kirsten B. Hornbeak, Lisa P. Jackson, Raena D. Sumiyoshi, Christopher L. Howerton, Sadie L. Hannah, Sonia Partap, Jennifer M. Phillips, Antonio Y. Hardan, Karen J. Parker

**Affiliations:** 1 Department of Psychiatry and Behavioral Sciences, Stanford University School of Medicine, Stanford, California, 94305, United States of America; 2 Department of Comparative Medicine, Stanford University School of Medicine, Stanford, California, 94305, United States of America; 3 Department of Pediatrics, Division of Pediatric Hematology, Oncology, SCT and Cancer Biology, Stanford University School of Medicine, Stanford, California, 94305, United States of America; 4 Department of Neurology and Neurological Sciences, Stanford University School of Medicine, Stanford, California, 94305, United States of America; Hamamatsu University School of Medicine, JAPAN

## Abstract

Brain arginine vasopressin (AVP) critically regulates normative social behavior in mammals, and experimental disruption of the AVP signaling pathway produces social impairments in rodent models. We therefore hypothesized that AVP signaling deficits may contribute to social impairments in children with autism spectrum disorder (ASD). Since blood measures (which are far easier to obtain than brain measures) of AVP are most meaningful if they are related to brain AVP activity, Study 1 tested the relationship between AVP concentrations in concomitantly collected blood and CSF samples from children and adults (N = 28) undergoing clinical procedures. Study 2 tested whether blood AVP concentrations: 1) differed between children with ASD (N = 57), their ASD discordant siblings (N = 47), and neurotypical controls (N = 55); and 2) predicted social functioning (using the NEPSY-II Theory of Mind and Affect Recognition tasks and the Social Responsiveness Scale) in this large, well-characterized child cohort. Blood AVP concentrations significantly and positively predicted CSF AVP concentrations (*F*_1,26_ = 7.17, *r* = 0.46, *p* = 0.0127) in Study 1. In Study 2, blood AVP concentrations did not differ between groups or by sex, but significantly and positively predicted Theory of Mind performance, specifically in children with ASD, but not in non-ASD children (*F*_1,144_ = 5.83, *p* = 0.017). Blood AVP concentrations can be used: 1) as a surrogate for brain AVP activity in humans; and 2) as a robust biomarker of theory of mind ability in children with ASD. These findings also suggest that AVP biology may be a promising therapeutic target by which to improve social cognition in individuals with ASD.

## Introduction

Autism spectrum disorder (ASD) is characterized by core deficits in social behavior and communication, and affects an estimated 1 in 68 U.S. children [1]. Despite being one of the most devastating childhood disorders, the basic biology of ASD remains poorly understood and there are no medications that treat the core social features of this disorder. Identifying the underlying pathophysiology of these core social deficits and developing novel treatments that specifically target these impairments are clearly important challenges.

The neurobiological systems critical for normative social functioning are arguably the most promising candidates by which to identify biomarkers of, and therapeutic targets for, ASD [2, 3]. Two such candidates are the neuropeptides oxytocin (OXT) and arginine vasopressin (AVP). OXT and AVP are primarily synthesized in the hypothalamus and released into both the brain via distributed neural pathways and systemic circulation via the posterior pituitary [4]. Extensive preclinical research has demonstrated the importance of both OXT and AVP biology in social functioning in multiple mammalian species [5–7]. Moreover, experimental dysregulation of these signaling pathways through pharmacological or genetic manipulation produces social impairments in rodents with relevance to ASD [8, 9].

Translation of these promising preclinical findings to patients with idiopathic ASD has focused almost exclusively on OXT therapy and endogenous OXT biology. Although single doses of intranasal OXT improve laboratory-based social measures in people with ASD [10, 11], longer-term ASD clinical trials have failed to provide convincing evidence for OXT efficacy over placebo treatment [12, 13]. Further, in the largest biomarker study published to date, blood OXT concentrations positively predicted social functioning in children with and without ASD [14], indicating that blood OXT concentrations are a biomarker of universal social functioning, and not specific to ASD.

Although less researched, there is compelling evidence that AVP biology may play a more important role in social functioning than previously appreciated. Central administration of selective AVPv1a receptor (AVPRv1a) antagonists, in the presence of normal brain OXT signaling, impairs social functioning in rodents [15, 16]. These pharmacological effects are especially evident in males, and given ASD’s male-biased prevalence, brain AVP peptide signaling deficits may be particularly relevant to understanding the risk for ASD.

Little is known about AVP biology as a biomarker of social deficits in people with ASD. This gap in knowledge is due, in part, to the difficulty of obtaining brain-relevant samples [e.g., cerebrospinal fluid (CSF)] from ASD patients in which to assess AVP concentrations, and the unknown relationship between blood (which is far easier to obtain) and brain sources of AVP. Several groups have nevertheless studied blood AVP concentrations in individuals with ASD. These groups have found that blood AVP concentrations are higher [17], lower [18], or do not differ [19], in individuals with ASD *vs*. controls. Relationships between blood AVP concentrations and social behavior in these studies were either not assessed or found to be unrelated. These studies also had various limitations including small study cohorts and use of nonstandard methods for ASD diagnosis and/or AVP measurement. Blood AVP concentrations likewise were not examined in ASD discordant siblings, despite the possibility that AVP concentrations could be related to the “broad autism phenotype”, in which relatives of individuals with ASD show subclinical impairments in social and biological functioning [20–22].

It therefore remains unknown whether blood AVP concentrations are related to CSF AVP concentrations, or whether AVP concentrations are related to ASD diagnosis or predict social functioning in people with ASD, their unaffected relatives, and/or neurotypical controls. The goals of this project therefore were to test whether blood AVP concentrations: 1) can serve as a surrogate for CSF AVP concentrations in humans; 2) differ between children with ASD, their ASD discordant siblings, and neurotypical controls; and 3) predict social functioning performance in a large, well-characterized child cohort.

## Materials and Methods

### Study 1

#### Participants

This research study, and its associated consent and assent forms, were reviewed and approved by the Stanford University Institutional Review Board. Twenty-eight pediatric and adult patients (15 F, 13 M) undergoing either clinically indicated lumbar punctures or other CSF-related procedures were recruited to this study. Participants were between four and 64 years of age (*M* = 17.17, *SEM* = 2.3) and included individuals of various ethnic backgrounds. Participant demographic and medical characteristics are provided in Table 1. Data from a subset of these patients has been outlined previously [23].

**Table 1:** Patient demographics and medical characteristics.

PATIENT NUMBER	CSF AVP (pg/mL)	BLOOD AVP (pg/mL)	SEX	AGE (YEARS)	ETHNICITY	TYPE OF ANESTHETIC	SAMPLE COLLECTION TIME	INDICATIONS FOR CSF PROCEDURE	DIAGNOSIS/PATHOLOGY REPORT
1	3.31	3.35	male	17.26	Caucasian	Local	14:27	Severe headache^1^	No abnormality determined
2	2.30	3.01	female	13.41	Caucasian	Local	18:30	Rule-out meningitis^1^	No abnormality determined
3	2.92	2.88	female	44.61	Asian	Local	15:43	Rule-out subarachnoid hemorrhage^1^	Subarachnoid hemorrhage
4	1.82	2.67	female	11.58	Hispanic	General	12:45	VP shunt tap^2^	Hydrocephalus
5	1.98	2.42	female	18.51	Caucasian	General	20:15	Pituitary abnormality^1^	Pituitary abnormality
6	5.66	9.19	female	23.81	Caucasian	Local	21:50	Breathing problem/hypotension^1^	No abnormality determined
7	3.78	2.46	male	64.38	Caucasian	Local	22:22	Rule-out pseudotumor cerebri^1^	No abnormality determined
8	3.16	10.96	female	15.48	Caucasian	Local	13:32	Maintenance chemotherapy^1^	Maintenance chemotherapy
9	2.33	3.47	male	11.22	Caucasian	General	09:15	Maintenance chemotherapy^1^	Maintenance chemotherapy
10	1.83	3.51	female	6.04	Asian	General	09:10	Maintenance chemotherapy^1^	Maintenance chemotherapy
11	2.27	5.85	male	21.26	Asian	General	12:10	Maintenance chemotherapy^1^	Maintenance chemotherapy
12	1.89	4.59	male	13.83	Caucasian	General	09:15	Maintenance chemotherapy^1^	Maintenance chemotherapy
13	2.14	3.12	female	14.16	Hispanic	General	09:50	Maintenance chemotherapy^1^	Maintenance chemotherapy
14	2.91	2.45	female	15.72	Caucasian	General	03:50	Maintenance chemotherapy^1^	Maintenance chemotherapy
15	1.74	3.31	female	5.86	Caucasian	General	10:45	Maintenance chemotherapy^1^	Maintenance chemotherapy
16	3.09	9.31	male	10.50	Asian	General	09:22	Maintenance chemotherapy^1^	Maintenance chemotherapy
17	2.17	2.14	female	13.20	Asian	General	10:22	Chiari craniotomy^3^	Chiari malformation
18	1.95	2.84	female	16.76	Asian	General	09:02	Maintenance chemotherapy^1^	Maintenance chemotherapy
19	1.70	3.01	female	8.65	African American	General	08:16	Maintenance chemotherapy^1^	Maintenance chemotherapy
20	2.18	9.01	male	24.09	Asian	General	14:05	Maintenance chemotherapy^1^	Maintenance chemotherapy
21	1.96	2.61	male	10.41	African American	General	08:56	Maintenance chemotherapy^1^	Maintenance chemotherapy
22	2.33	4.85	male	15.37	Asian	General	09:34	Maintenance chemotherapy^1^	Maintenance chemotherapy
23	2.70	3.93	male	18.24	Hispanic	General	10:30	Maintenance chemotherapy^1^	Maintenance chemotherapy
24	2.77	2.81	female	4.01	Asian	General	09:30	Maintenance chemotherapy^1^	Maintenance chemotherapy
25	1.56	2.69	female	15.81	Asian	General	09:18	Induction chemotherapy^1^	Induction chemotherapy
26	2.19	6.22	male	15.33	Hispanic	General	11:41	Chiari craniotomy^3^	Chiari malformation
27	3.30	8.74	male	5.98	Hispanic	General	08:13	Maintenance chemotherapy^1^	Maintenance chemotherapy
28	3.58	6.66	male	25.40	Caucasian	Local	21:44	Unexplained change in mental state^1^	No abnormality determined

Abbreviations: AVP, arginine vasopressin; CSF, cerebrospinal fluid; ALL, acute lymphoblastic leukemia; AML, acute myeloblastic leukemia

^1^indicates CSF collected from lumbar puncture

^2^indicates CSF collected from left ventricle

^3^indicates CSF collected from the cisterna magna.

Participants were recruited from Stanford Hospital & Clinics (SHC) and the Lucile Packard Children’s Hospital (LPCH). Clinical indications for blood and CSF collection included rule-out diagnoses (i.e., clinical assessment to eliminate from consideration the possible presence of a condition or disease including pseudo-tumor cerebri, meningitis, and subarachnoid hemorrhage), headaches, ventriculoperitoneal shunt taps, craniectomies, and blood/tissue diseases such as leukemia that required CSF access in diagnosis or treatment. Patients scheduled for these clinical procedures were identified by health care providers as potential study participants. All patients were research consented prior to sample collection (i.e., in addition to the medical consent obtained by health care providers for the standard of care procedure). Adult participants (i.e., those 18 years or older) were consented in writing. Parents and/or legal guardians of pediatric participants (i.e., children under 18 years of age) provided written consent. If the child was deemed intellectually capable of understanding the study (i.e., those aged 7 to 17 years), written assent was also obtained from the child participant.

CSF aliquots for this study were either provided as an additional amount to the volume acquired for clinical purposes or reserved at the time of clinical procedure in lieu of disposal.

Inclusion criteria for participants consisted of clinically indicated reason for CSF collection and participant willingness to undergo blood collection, English speaking, and between 18 months and 99 years of age. Exclusion criteria for participants consisted of pregnancy at the time of biological sample collection.

#### CSF and blood sample collection and processing procedures

CSF was obtained for research purposes using standard sterile procedures by clinical staff following administration of either local or general anesthetic. CSF was collected from the lumbar region in the majority (*N* = 25) of patients while a small number of patients had CSF collected from rostral regions including the left ventricle (*N* = 1) and the cisterna magna (*N* = 2). CSF samples were immediately aliquoted into siliconized polypropylene tubes and flash-frozen on dry ice. Whole blood was collected (at the same time as CSF) into chilled EDTA-treated vacutainer tubes from a central or arterial line and placed on wet ice. Whole blood samples were promptly centrifuged (1600×*g* at 4°C for 15 min), the plasma fraction aliquoted into siliconized polypropylene tubes, and flash-frozen on dry ice. All samples were stored at -80°C until quantification for AVP concentrations.

#### Sample preparation and AVP quantification

CSF and blood AVP concentrations were quantified using a commercially available enzyme immunoassay kit (Enzo Life Sciences, Inc., Farmingdale, NY). This kit has been validated for use in humans and is highly specific and selectively recognizes AVP and not related peptides. Per the technological division of Enzo Life Sciences, the cross-reactivity with oxytocin is <0.001% and the minimum assay sensitivity is 3.39 pg/mL. A trained technician blinded to clinical conditions performed sample preparation and AVP quantification following established procedures. CSF samples (800 μL/participant) were thawed in an ice bath and then mixed with an equal volume of ice-cold acetone, briefly vortexed, and centrifuged at 1°C for 15 min at 4,000×*g*. Samples were next evaporated at RT using compressed nitrogen and then reconstituted in 240 μL assay buffer prior to AVP quantification. Plasma samples (1 mL/participant) were extracted using the solvent method recommended by the manufacturer. Briefly, equal volumes of 40:60 butanol:diisopropyl ether were added to samples prior to centrifugation at RT for 5 min at 8,000×*g*. The top organic layer was discarded and the aqueous solution transferred to a new microcentrifuge tube. A 2:1 volume of ice cold acetone was added to all samples prior to centrifugation at 4°C for 20 min at 12,000×*g*. The supernatant was then transferred to 15 mL Falcon tubes and a volume of 5:1 ice cold petroleum ether was added. Samples were briefly vortexed, centrifuged at 1°C for 10 min at 3350×*g*, and the top ether layer discarded. Samples were then evaporated at RT using compressed nitrogen. Each evaporated CSF and plasma sample was reconstituted in 250 μL of assay buffer prior to AVP quantification to provide sufficient sample volume to run each participant’s samples in duplicate wells (100 μL per well). Given the sensitivity limitations of the commercial assay, this ensured that the plated samples contained high enough quantities of AVP to be read above the limit of detection. The program used to calculate pg/mL concentrations of AVP allows for extrapolation based on the sample concentration factor. That is, the program extrapolates the final AVP concentrations by dividing the results by the fold-difference in original sample volume. This method, which has been validated in our and other laboratories [14, 23, 24], and is used widely in this research field, increases the analyte’s concentration in each well. This procedure ensures that each sample (when it is read) falls within the linear portion of the standard curve, above the assay’s limit of detection. All samples were assayed in duplicate with a tuneable microplate reader for 96-well format at 405nm with correction at 570nm, according to manufacturer’s instructions. All standards were run in triplicate and provided intra- and inter-assay coefficients of variation below 10%.

### Study 2

#### Participants

This research study, and its associated consent and assent forms, were reviewed and approved by the Stanford University Institutional Review Board. Fifty-seven children with ASD (*N* = 9 F, 48 M), 47 ASD discordant siblings (*N* = 20 F, 27 M), and 55 unrelated neurotypical control children (*N* = 19 F, 36 M) between the ages of 3 and 12 years were recruited to participate in this study. Detailed participant characteristics are presented in Table 2. Parents and/or legal guardians of these pediatric participants provided written consent prior to the initiation of experimental procedures. If the child was deemed intellectually capable of understanding the study (i.e., those aged 7 to 12 years), written assent was also obtained from the child participant.

**Table 2:** Participant characteristics.

Participants	Sex*			Ethnicity*					
	*N*	Female	Male	Caucasian	Asian	Other	Age*	Full-scale IQ*	Blood collection time, min^ns^
ASD									
Autistic	29	3	26	15	7	7	7.92 ± 0.45^ab^	83.55 ± 3.53^a^	12:25 PM ± 15.98
PDD-NOS	28	6	22	22	2	4	9.25 ± 0.44^a^	99.79 ± 4.00^b^	12:18 PM ± 14.14
Sibling	47	20	27	24	15	8	7.89 ± 0.43^ab^	109.18 ± 1.84^c^	12:38 PM ± 9.42
Control	55	19	36	41	3	11	7.31 ± 0.41^b^	115.60 ± 1.30^c^	12:33 PM ± 7.38

**χ** ^2^ was used to test whether the distribution of individuals to different groups differed by sex and by ethnicity. Significant effects were found for each. However, post hoc tests failed to find any group that showed a significant difference from expected (by sex or by ethnicity). For age, full-scale IQ, and blood collection time, differences between groups were tested with a simple one-way general linear model. The values are expressed in mean ± SEM. Abbreviations

* = P < 0.05

^ns^ = not significant.

Values with different letter superscripts (i.e., ^a^, ^b^, or ^c^) within the same column of the table differ significantly, whereas values with the same letter superscript (i.e., ^a^, ^b^, or ^c^) within the same column of the table do not differ, according to Tukey’s post hoc test.

Children with ASD and their siblings were primarily recruited through the Autism and Developmental Disorders Research Registry, and by flyers posted in the Autism and Developmental Disorders Clinic, at Stanford University. Unrelated control participants were recruited through advertisements posted online (e.g., parent listservs, craigslist.org) or hardcopy in the surrounding community (e.g., pediatrician offices, shopping malls). All participants were: 1) pre-pubertal; 2) in good medical health; 3) willing to provide a blood sample; and, 4) were capable of completing all behavioral testing. Participants with ASD were included if they had a full scale IQ of 50 and above. Control participants and siblings were included if they had an IQ in the average range. Cognitive functioning was determined using the Stanford Binet 5th Edition [25]. Participants were selected from a larger study cohort published elsewhere [14] on the basis that they had completed all behavioral assessments.

Children with a diagnostic history of ASD underwent a comprehensive diagnostic evaluation to determine the accuracy of the previous diagnosis based on DSM-IV-TR criteria, which was confirmed with research diagnostic methods. These diagnostic instruments included the Autism Diagnostic Instrument-Revised (ADI-R) [26, 27] and the Autism Diagnostic Observation Schedule (ADOS) using the revised algorithms [28–30], and were performed by research staff trained by a research-reliable clinician. Although all participants met DSM-IV-TR criteria for ASD, expert clinical opinion and scores on the ADI-R were also used to characterize children with ASD as having autistic disorder or pervasive developmental disorder-not otherwise specified (PDD-NOS). Children with ASD who met DSM-IV-TR criteria diagnosis of autistic disorder and scored above the cut-off for autistic disorder on ADI-R and ADOS were categorized as having autistic disorder. Children with ASD who met DSM-IV-TR criteria diagnosis of autistic disorder and scored above the cut-off for autistic disorder on ADI-R but in the autism spectrum range on the ADOS were categorized as having PDD-NOS. Exclusion criteria included: 1) a genetic, metabolic, or infectious etiology for ASD on the basis of medical history, neurologic history, and available laboratory testing for inborn errors of metabolism and chromosomal analysis; and, 2) seizures or a DSM-IV-TR diagnosis of any severe mental disorder such as schizophrenia and bipolar disorder. Participants taking psychotropic medications were included as long as their medications were stable. Siblings of children with ASD were required to have no evidence of ASD on the basis of clinical evaluation and research diagnostic assessments. They were also required to have no present or past history of any severe neuropsychiatric disorder such as schizophrenia or bipolar disorder on the basis of a clinical psychiatric evaluation and information obtained from behavioral scales. Neurotypical control children were required to: 1) be free of neurological disorders in the present or past on the basis of history; 2) be free of psychiatric disorders in the present or past on the basis of information obtained from behavioral scales, a clinical psychiatric evaluation, and if needed, the Kiddie-Schedule for Affective Disorders and Schizophrenia for School-Aged Children [31]; 3) have no historical evidence of significant difficulty during mother’s pregnancy, labor, delivery, or in the immediate neonatal period, or abnormal developmental milestones based on neurological history; and, 4) have no sibling diagnosed with ASD.

#### Social phenotyping

Social phenotyping included the following instruments. The NEPSY-II [32] is a norm-referenced measure of child neurocognitive abilities. The NEPSY-II Social and Perception Domain: Affect Recognition and Theory of Mind tasks were the only subscales used in this study. The Social Responsiveness Scale (SRS) is a norm-referenced questionnaire that measures social behavior in both clinical and non-clinical populations [33]. It includes five sub-scales: social awareness, social cognition, social communication, social motivation, and autistic mannerisms. The psychometric properties have been tested in younger and older participants, and the SRS Total score (used in this study) is continuously distributed within each group.

#### Blood sample collection and processing procedures

Fifteen mL of whole blood was drawn from the child’s antecubital region by a trained phlebotomist using standard protocols at the LPCH outpatient laboratory facility between 10 AM and 2 PM. Blood was collected into chilled EDTA-treated vacutainer tubes and immediately placed on wet ice. Whole blood was promptly centrifuged (1300×*g* at 4°C for 10 minutes), the plasma fraction aliquoted into polypropylene tubes and flash-frozen on dry ice, and stored at -80°C.

#### Sample preparation and AVP quantification

Sample extraction and concentration procedures were initiated by thawing plasma samples in an ice bath. Waters Sep-Pak C18 columns (Waters Corp., Milford, MA) were conditioned with 1 mL of HPLC grade methanol followed by 1 mL of molecular biology grade water. Plasma samples (1 mL/participant) were drawn through the column by vacuum on a Supelco SPE vacuum manifold (Sigma-Aldrich Group, Bellefonte, PA). The columns were washed with 1 mL of wash buffer (89:10:1 water:acetonitrile:TFA) followed by 1 mL of elution buffer (80:20 acetonitrile:water). Elutes were evaporated at RT using compressed nitrogen, and reconstituted in assay buffer prior to quantification. Samples were assayed in duplicate and AVP concentrations quantified as described above in Study 1. Assays were performed by a technician blinded to experimental conditions. Intra- and inter-assay coefficients of variation were <10%.

### Statistical analysis

Data were managed using REDCap [34] and analyzed using JMP V.10 (SAS Institute Inc., Cary, NC). In Study 1, the relationship between blood and CSF AVP concentrations was assessed using a general linear model (GLM), which included the following blocking factors: age, sex, ethnicity, sample collection time, and type of anesthetic treatment. These blocking factors were selected by the research team as the most likely to contribute extraneous sources of variability. To correct for the blocking factors in the analysis, the regression line is partialed (controlled) for other variables in the analysis, and calculated at the mean value of those variables. The assumptions of GLM (normality of error, homogeneity of variance, and linearity) were examined graphically and suitable transformations applied as required.

In Study 2, we first used a GLM to test for differences in mean blood AVP concentrations and social functioning by group and sex (while blocking for age, ethnicity, blood sample collection time, and full scale IQ). We represented group by subdividing (nesting) this variable into ASD (autistic and PDD-NOS) vs. non-ASD (sibling and neurotypical control) individuals. This allowed us to test explicitly whether any overall difference was due to differences between ASD and non-ASD individuals in general, or to a particular group. (This approach is far more statistically powerful than resorting to post-hoc multiple comparisons between groups.) Second order interactions between group and sex were also tested for blood AVP concentrations. Next we analyzed the relationship between each of the social behavior measures (i.e., Theory of Mind, Affect Recognition, and SRS Total scores) and blood AVP concentrations in separate GLM models. Experimental factors for each model included blood AVP concentrations and group (represented as group-nested-within-diagnosis). Second order interactions between all experimental factors were included. Blocking factors were sex, age, ethnicity, blood sample collection time, and full scale IQ. The assumptions of GLM (normality of error, homogeneity of variance, and linearity) were examined graphically and suitable transformations applied as required. Significant interactions were examined with post-hoc Bonferroni-corrected orthogonal planned contrasts.

## Results

### Study 1

**Fig 1:**
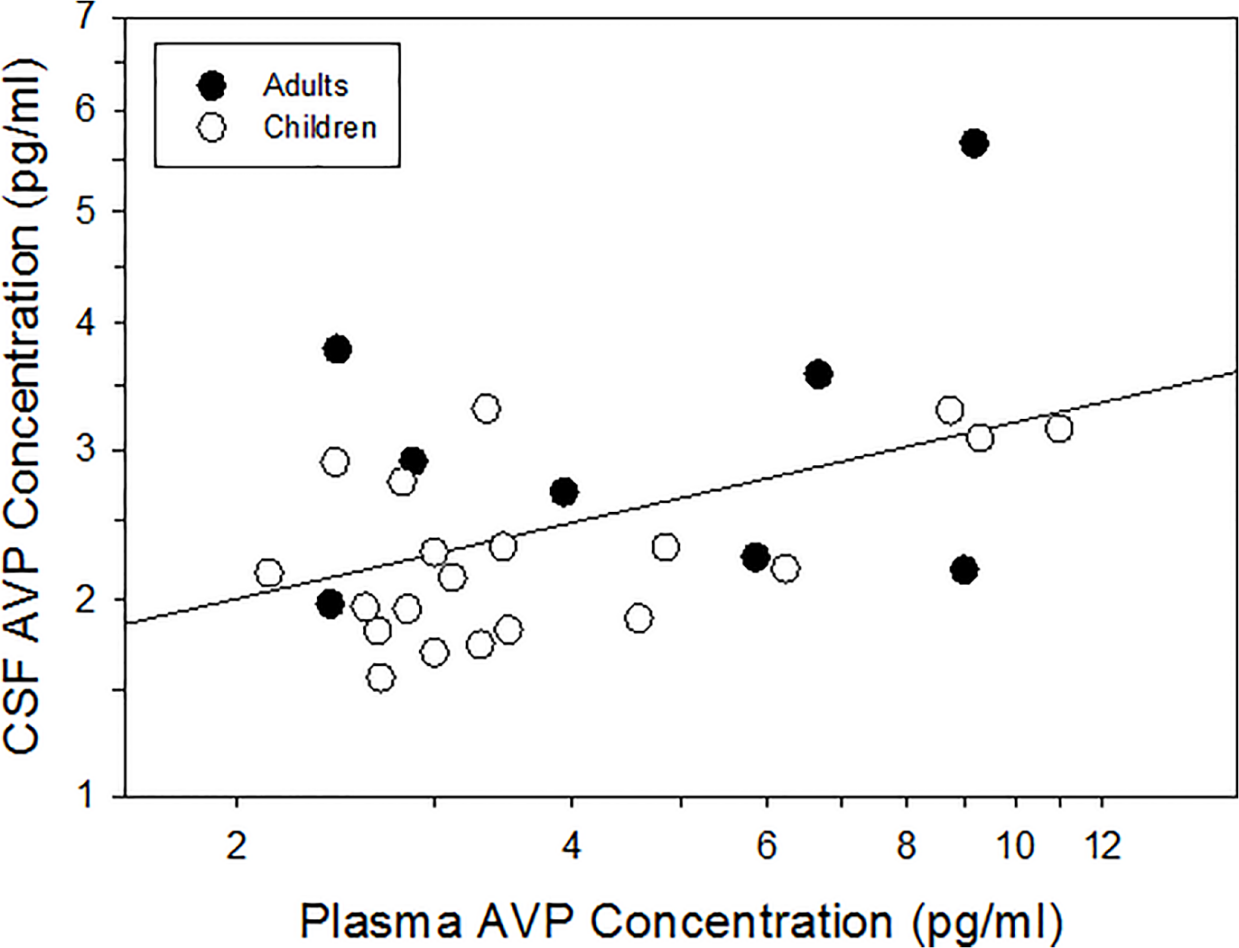
Blood arginine vasopressin (AVP) concentration significantly and positively predicts cerebrospinal fluid (CSF) AVP concentration. Sample size is *n* = 28.

In line with our recent study of neonatal humans [24], we found that blood AVP concentrations significantly and positively predicted CSF AVP concentrations (*F*_1,26_ = 7.17, *r* = 0.46, *p* = 0.0127; Fig 1) in this independent, older cohort. This significant relationship was maintained after controlling (i.e., blocking) for extraneous sources of variability (*F*_1,21_ = 5.08, *r* = 0.44, *p* = 0.035), and these variables (i.e., age, sex, ethnicity, sample collection time, and type of anesthetic) each had no significant effect. Importantly, the significant and positive relationship between AVP concentrations in blood and CSF was maintained (*F*_1,18_ = 2.75, *r* = 0.54, *p* = 0.0131) when only child participants (*<*18 years; *n* = 20) were included in the analysis.

### Study 2

**Fig 2:**
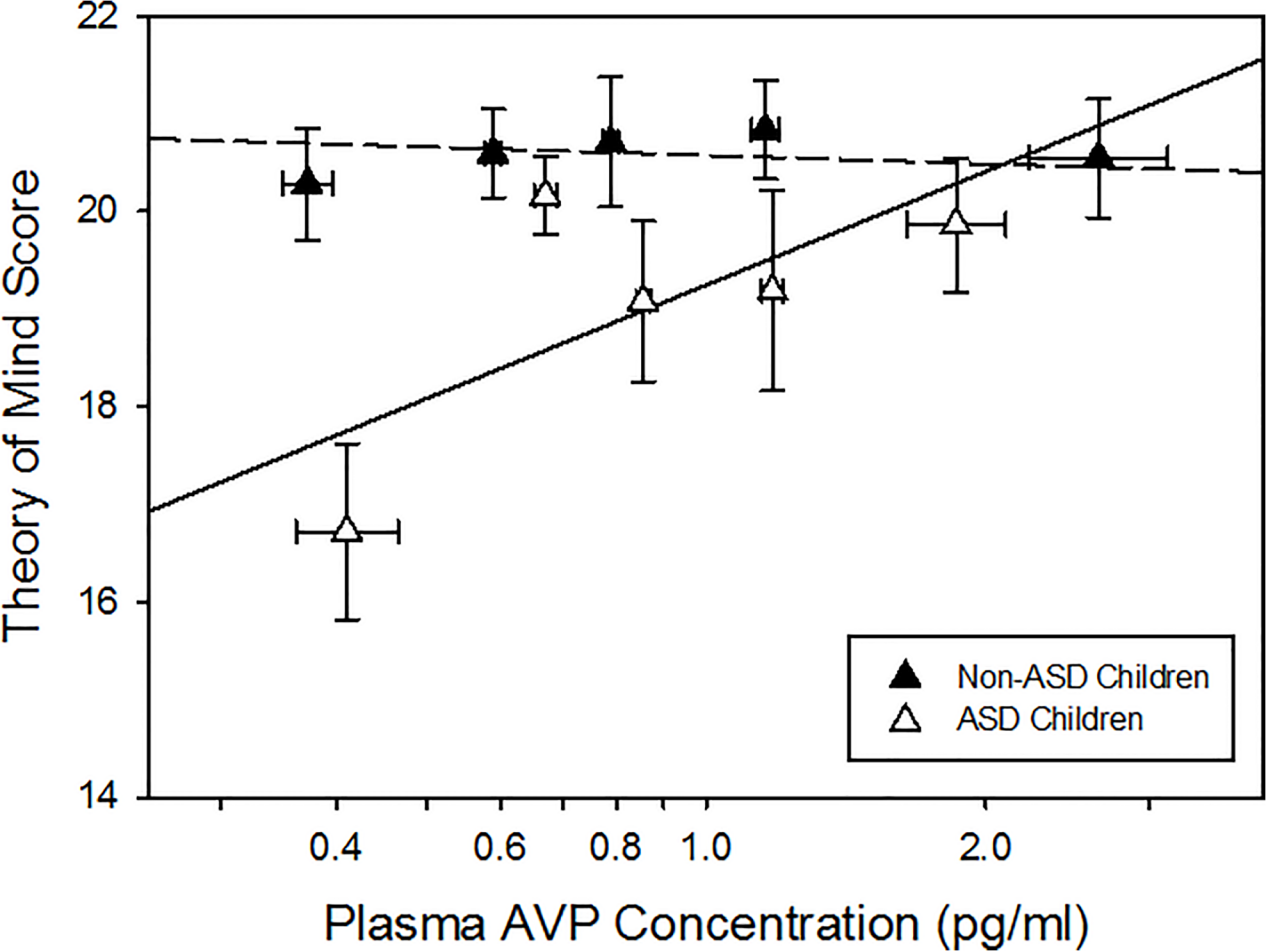
Blood AVP concentration predicts NEPSY Theory of Mind score in ASD children (autistic and PDD-NOS) but not in non-ASD children (sibling and neurotypical control). Data have been corrected for the following blocking factors: age, sex, ethnicity, blood sample collection time, and full scale IQ. Data are plotted as a mean and standard error for each AVP quintile within the ASD and non-ASD groups. The means shown are of the log transformed plasma AVP values used in the analysis itself. ASD Quintile (Q) Q1 *n* = 11, Q2 *n* = 12, Q3 *n* = 11, Q4 *n* = 11, Q5 *n* = 12; Non-ASD Q1 *n* = 20, Q2 *n* = 21, Q3 *n* = 21, Q4 *n* = 19, Q5 *n* = 21.

In contrast to previous pilot studies [17, 18], and after controlling for possible extraneous sources of variability (i.e., age, ethnicity, blood sample collection time, full scale IQ; none of which were significant themselves), blood AVP concentrations did not differ by group or sex, nor were any interaction effects (i.e., group x sex) observed. As expected, the child ASD group exhibited social impairments on all three measures compared to the neurotypical control and ASD discordant sibling groups, which did not differ from one another [35]. The relationship between blood AVP concentrations and Theory of Mind score did, however, differ between children with and without ASD (*F*_1,144_ = 5.83, *p* = 0.017; Fig 2). This relationship did not differ between children with autistic disorder and PDD-NOS *versus* sibling and control subgroups (*F*_2,144_ = 0.07, *p* = 0.933), confirming that these subgroups could be combined into the larger ASD and non-ASD groups used in all subsequent analyses. Post hoc analysis showed that there was no relationship between AVP concentrations and Theory of Mind score in non-ASD children (*F*_1,144_ = 0.12, *p* = 0.723), but blood AVP concentrations significantly and positively predicted Theory of Mind score in children with ASD (*F*_1,144_ = 6.50, *p* = 0.0118, Bonferroni-corrected critical alpha = 0.025). The relationship between blood AVP concentrations and social cognition was specific to Theory of Mind performance, as blood AVP concentrations did not predict Affect Recognition score or SRS Total score in either the ASD or non-ASD group.

## Discussion

Our collective findings demonstrate that blood AVP concentrations can be used: 1) as a surrogate for brain AVP activity in humans (Study 1; Fig 1); and 2) as a biomarker of Theory of Mind ability in children with ASD (Study 2; Fig 2). The discovery of a robust blood-based biomarker of social functioning in ASD is particularly important given that invasive (e.g., lumbar puncture) measures of brain activity are unlikely to be used in routine clinical settings. These findings also suggest that AVP signaling impairments might be a promising target for drug development, particularly in a subset of ASD individuals with the most impaired Theory of Mind scores.

There has been considerable scientific interest in assessing relationships between peripheral (i.e., blood, urine, saliva) neuropeptide concentrations and complex social functioning in neurotypical and clinical populations. Research on humans has occurred, however, in the absence of compelling evidence that peripheral assessments of neuropeptide concentrations are related to brain neuropeptide activity. Study 1 bridged this important gap in knowledge by providing empirical evidence that blood AVP concentrations significantly and positively (*r* = 0.46) predict CSF AVP concentrations in humans, aged 4 to 64 years. These findings also extend our recent study which documented the same relationship in neonatal infants sampled within 72 hours of birth [24]. Considered collectively, findings from these two scientific reports indicate that measurement of AVP in blood samples is a valid tool for inferential assessment of brain AVP activity, and that the predictive relationship between blood and CSF AVP concentrations likely extends across the human lifespan.

Blood-based biomarkers that improve our understanding of ASD’s social phenotypic heterogeneity will enhance diagnostic accuracy and provide an objective metric for treatment response. Although single-doses of intranasal AVP administered to neurotypical individuals enhance memory for happy and angry faces [36], improve recognition of positively and negatively valenced social words [37], and increase neural activity in known AVP brain circuitry during a laboratory-based cooperation task [38], no published clinical trials have yet evaluated the efficacy of intranasal AVP to improve social functioning in people with ASD. Results from Study 2 nevertheless provide valuable direction for future AVP pharmacotherapy in ASD patients. Specifically, *a priori* stratification of participants in intranasal AVP treatment trials on the basis of known blood AVP signaling deficits may enhance: 1) assessment of the therapeutic potential of AVP to improve theory of mind performance and other dimensions of social functioning; and 2) identification of ASD patients most likely to benefit from AVP treatment.

The promise of therapeutically enhancing brain AVP signaling in people with ASD is underscored by recent findings from the first neuropeptide receptor mapping study of postmortem primate brain tissue [39]. This study revealed that AVPRv1a are widely distributed throughout the extended neural amygdala, suggesting that exogenously administered AVP will be able to target directly neural pathways known to regulate social functioning. Interesting, OXT receptors were not present in characteristic “social” brain regions, but rather, restricted to several hind brain regions largely involved in early visual and auditory processing. These receptor distribution data may also explain the efficacy of single-doses of OXT to enhance eye gaze to social cues in individuals with ASD and related disorders [11, 40], and the equivocal findings of longer-term OXT treatment trials aimed at enhancing complex social cognition [12, 13]. Although AVP and OXT can bind to each other’s receptors at sufficiently high concentrations, these neuropeptide receptor mapping and pharmacological data nevertheless raise the provocative question as to whether AVP, rather than OXT, pharmacotherapy holds the most promise for effectively improving social functioning in patients with ASD.

The relationship between blood AVP concentrations and Theory of Mind performance was specific to individuals with ASD. Exactly why non-ASD individuals did not show this relationship is unknown, but there are several possibilities. For example, there may indeed be a relationship between AVP and theory of mind ability in unaffected individuals, but “ceiling” effects in performance on this particular measure may have obscured this relationship. Alternatively, individual differences in AVP concentrations may only begin to deleteriously affect theory of mind ability at the lower end of the functional range, which is why this relationship is only evident in affected individuals. Unfortunately, the available data do not help differentiate between these two possibilities. Follow up studies that: 1) assess blood AVP concentrations in ASD discordant siblings who *do* exhibit subclinical social deficits in NEPSY theory of mind performance; and/or 2) employ a theory of mind assessment instrument that produces a broad range of scores even in neurotypical individuals are now required to answer this question.

There are several limitations of the present studies. CSF was collected in Study 1 in a sample of convenience: A heterogeneous population of patients undergoing clinically indicated (and standard of care) CSF sample collection for a variety of medical conditions. Although we found no relationship between AVP concentrations and medical condition, it remains possible that the relationship between blood and CSF AVP concentrations nevertheless could differ in medically healthy children. In Study 2, we were able to obtain only a single blood sample from each child participant due to ethical considerations of this invasive procedure. Future research therefore is needed to test for trait-like consistency in blood AVP concentrations in animal models and/or in adult humans. In closing, despite these potential limitations, the present findings provide the first evidence for AVP as a blood-based biomarker of social functioning in, and as a promising therapeutic target for, children with ASD.
